# Pericardial Fat and Right Ventricular Morphology: The Multi-Ethnic Study of Atherosclerosis- Right Ventricle Study (MESA-RV)

**DOI:** 10.1371/journal.pone.0157654

**Published:** 2016-06-16

**Authors:** David S. Wenger, Steven M. Kawut, Jingzhong Ding, David A. Bluemke, Catherine L. Hough, Richard A. Kronmal, Joao A. Lima, Peter J. Leary

**Affiliations:** 1 Department of General Internal Medicine, University of Washington, Seattle, Washington, United States of America; 2 Department of Medicine and Epidemiology, Division of Pulmonary and Critical Care Medicine, Perelman School of Medicine at the University of Pennsylvania, Philadelphia, Pennsylvania, United States of America; 3 Department of General Internal Medicine, Wake Forest University, Winston-Salem, North Carolina, United States of America; 4 Department of Radiology, Division of Radiology and Imaging Sciences, NIH Clinical Center, Bethesda, Maryland, United States of America; 5 Department of General Internal Medicine, Division of Pulmonary and Critical Care, University of Washington, Seattle, Washington, United States of America; 6 Department of Biostatistics, University of Washington, Seattle, Washington, United States of America; 7 Department of Radiology, Johns Hopkins University, Baltimore, Maryland, United States of America; Indiana University, UNITED STATES

## Abstract

**Background:**

Pericardial fat has been implicated in the pathogenesis of obesity-related cardiovascular disease. Proposed mechanisms may be relevant in right heart failure, but relationships between pericardial fat and right ventricular (RV) morphology have not been explored.

**Methods:**

The Multi-Ethnic Study of Atherosclerosis is a prospective cohort that enrolled participants without clinical cardiovascular disease. Pericardial fat was measured using computed tomography and RV parameters using cardiac MRI. Linear regression estimated associations of pericardial fat with RV mass, RV end diastolic volume (RV-EDV), RV end systolic volume (RV-ESV), RV stroke volume (RV-SV), and RV ejection fraction (RV-EF). Limited models adjusted for age, gender, race, height, and study site with and without weight. Fully adjusted models also accounted for socioeconomic parameters and health behaviors. Adjustment for left ventricular morphology, metabolic syndrome, and systemic inflammation was also performed.

**Results:**

The study sample included 3988 participants with complete assessment of RV morphology, pericardial fat and all covariates. Greater pericardial fat volume was associated with reduced RV mass (-0.3g per 40 cm^3^ increase in pericardial fat, p<0.001), smaller RV-EDV (-3.7ml per 40 cm^3^ increase in pericardial fat, p<0.001), smaller RV-ESV (-1.0ml per 40cm^3^ increase in pericardial fat, p<0.001), and smaller RV-SV (-2.7mL per 40 cm^3^ increase in pericardial fat, p<0.001) in participants after adjustment for weight. Associations were unchanged when accounting for health behaviors, markers of systemic inflammation, and the metabolic syndrome.

**Conclusions:**

Greater pericardial fat was associated with reduced RV mass, smaller RV-EDV, smaller RV-ESV, and smaller RV-SV in participants after adjustment for weight. Relationships between pericardial fat and RV morphology could be relevant to diseases of right heart failure.

## Introduction

Pericardial fat is ectopic adipose tissue surrounding the heart. It is more prominent in many cardiovascular diseases and has been implicated in the pathogenesis of coronary atherosclerotic disease, atrial fibrillation, and left heart failure [1–3]. The relationship between pericardial fat and cardiovascular pathology is controversial. Some feel that pericardial fat functions merely as a surrogate marker of systemic adiposity, while others suggest that due to its anatomic location and the lack of a fascial plane delineating it from the heart, pericardial fat is as a direct mediator of disease [3,4].

While the association between pericardial fat and left heart disease is established, relationships with the right heart are unknown. Obesity, insulin resistance, and the metabolic syndrome, conditions associated with increased pericardial fat, have been linked to poor RV myocardial adaptation [5]. Mechanistic studies suggest dysregulated inflammation may promote pathologic remodeling [6] and we have previously shown that systemic inflammation is associated with lower RV mass and smaller RV volumes in MESA participants [7]. Similar to visceral adipose tissue, pericardial fat secretes both pro- and anti- inflammatory chemokines, and its location may allow pericardial fat to uniquely influence myocardial inflammation [8,9].

We therefore sought to examine the relationship between computed tomography (CT) measures of pericardial fat and magnetic resonance imaging (MRI) measures of RV morphology in a multiethnic cohort of adults free of clinical cardiovascular disease. We hypothesized that more pericardial fat would be independently associated with reduced RV mass, smaller end-diastolic volume (EDV), smaller end-systolic volume (ESV), smaller stroke volume (SV), and lower ejection fraction (EF).

## Methods

The Multi-Ethnic Study of Atherosclerosis (MESA) is a multicenter prospective cohort that was designed to investigate subclinical cardiovascular disease in white, African-American, Hispanic, and Chinese subjects [10]. In 2000–2002, MESA recruited 6,814 subjects aged 45–84 years from six U.S. communities. Exclusion criteria included clinical cardiovascular disease, weight greater than 300 lbs, pregnancy, or impediment to long-term participation. The institutional review boards of all collaborating institutions approved the protocols of MESA and the studies described herein. The MESA-Right Ventricle Study, which includes the current investigation, was an ancillary study to interpret RV morphology in a large subset of MESA participants.

### Cardiac magnetic resonance imaging measures

The cardiac MRI protocol and methods of interpretation of left ventricular (LV) and RV parameters have been previously reported [11,12]. Briefly, endocardial and epicardial borders of the ventricles were traced on short-axis cine images at end-diastole and end-systole. Papillary muscles and trabeculae were included in ventricular volumes, but excluded from ventricular mass [13]. The difference between the epicardial and endocardial volumes at end-diastole multiplied by the specific gravity of the heart (1.05 g/cm^3^) was used to calculate ventricular mass [11]. SV was calculated by subtracting the ESV from the EDV. EF was calculated by dividing SV by EDV. The protocol included blinded rereads by the same reader. The intra-reader intra-class correlation coefficient (ICC) for RV mass was 0.94 (229 scans), for RV-EDV was 0.99 (230 scans), for RV-ESV was 0.99 (230 scans), and for RV-EF was 0.89 (230 scans). In addition, blinded rereads by a second reader were performed. The inter-reader ICC on 240 scans for RV mass, RV-EDV, RV-ESV, and RV-EF was 0.89, 0.96, 0.96, and 0.80, respectively [12].

### Pericardial fat

The pericardial fat CT protocol has been described previously [14]. Pericardial fat volume was measured from four simultaneous 2.5 mm cardiac sections captured by cardiac CT using electrocardiography triggering. Pericardial fat was defined as epicardial fat within the visceral pericardium plus paracardial (e.g. mediastinal) fat [15]. The pericardial fat volume was measured on sections from the region of the heart within 15 mm above and 30 mm below the superior extent of the left main coronary artery [16]. This selected region includes the pericardial fat around the left main coronary artery and the proximal portions of the left anterior descending and right coronary arteries. The anterior border of the volume was defined by the chest wall, and the posterior border was defined by the aorta and the bronchus. The pericardial fat volume (in cubic centimeters) was measured by using Volume Analysis software (GE Healthcare, Waukesha, Wis), with an attenuation threshold of -190 to -30 Hounsfield Units used to identify fat-containing voxels. The final volume was the sum of all voxels containing pericardial fat. The increment of analysis was 40 cm^3^, which is approximately one standard deviation difference in pericardial fat in the MESA cohort.

### Biomarkers and Covariates

Inflammatory plasma biomarkers were measured using fasting blood samples at the baseline MESA exam by the Laboratory for Clinical Biochemistry Research at University of Vermont (Burlington, VT). C-reactive Protein (CRP) was measured using the BNII nephelometer (N High Sensitivity CRP; Dade Behring Inc., Deerfield, IL). Coefficients of variation (CV) for CRP ranged from 2.1 to 5.7%. Interleukin-6 (IL-6) was measured by ultra-sensitive ELISA (Quantikine HS Human IL-6 Immunoassay; R&D Systems, Minneapolis, MN). CV for IL-6 was 6.3%.

Other covariates including age, gender, race/ethnicity, height, weight, waist and hip circumference, smoking status, pack years, weekly exercise performance, educational achievement, income, and the presence of hypertension, hyperlipidemia, or glucose intolerance were assessed at the initial MESA exam, are described in the MESA Manual of Operations (http://www.mesa-nhlbi.org/manuals.aspx), and were chosen *a priori* on the basis of known associations with ventricular size, heart disease, and comorbities. Metabolic syndrome was defined as the presence of 3 or more of the following clinical criteria: (1) Waist circumference >102cm for men or >88cm for women, (2) triglycerides >150 mg/dL, (3) high density lipoproteins (HDL) < 40mg/dl for men or <50 mg/dl for women, (4) blood pressure ≥ 135/85mmHg, (5) fasting plasma glucose ≥ 110 mg/dl (National Cholesterol Education Program (NCEP) Adult Treatment Panel III Guidelines) [17].

### Statistical Analysis

We used linear regression to characterize the relationship between pericardial fat and RV parameters. In limited models, we adjusted for age, race/ethnicity, height, and study site. Because of its importance to the association of interest, weight was added separately to a second model with limited adjustment and included in all subsequent models.

In fully adjusted models, we also included participants’ education, income, and cardiovascular risk factors including intentional exercise, smoking status, and number of pack years. Separate models further adjusted for LV morphology. Adjustment for the corresponding LV parameter was used to evaluate observed associations for independence from LV morphology (e.g., increased LV mass causing pulmonary venous hypertension leading to increased RV mass) and to better account for differences in body size. Because RVSV and LV stroke volume are interdependent, we adjusted for LV mass in this case.

In fully adjusted exploratory models, we evaluated whether observed associations could be accounted for by the presence or absence of the metabolic syndrome or differences in systemic inflammatory markers (CRP and IL-6). The presence or absence of the metabolic syndrome reduces differences in waist circumference, triglycerides, HDL, blood pressure, and fasting plasma glucose to a single binary composite variable. Using this approach, we were concerned that residual confounding imposed by components of the metabolic syndrome might exist. To minimize this risk we performed a sensitivity analysis of the fully adjusted model with further adjustment by the components of metabolic syndrome modeled as continuous variables. Further exploratory models evaluated whether age, gender, the presence of diabetes or the metabolic syndrome were effect modifiers of the association between pericardial fat and RV morphology. Statistical significance was defined as P <0.05. Analyses were performed with STATA 13.0 (Stata-Corp, College Station, TX).

## Results

**Fig 1:**
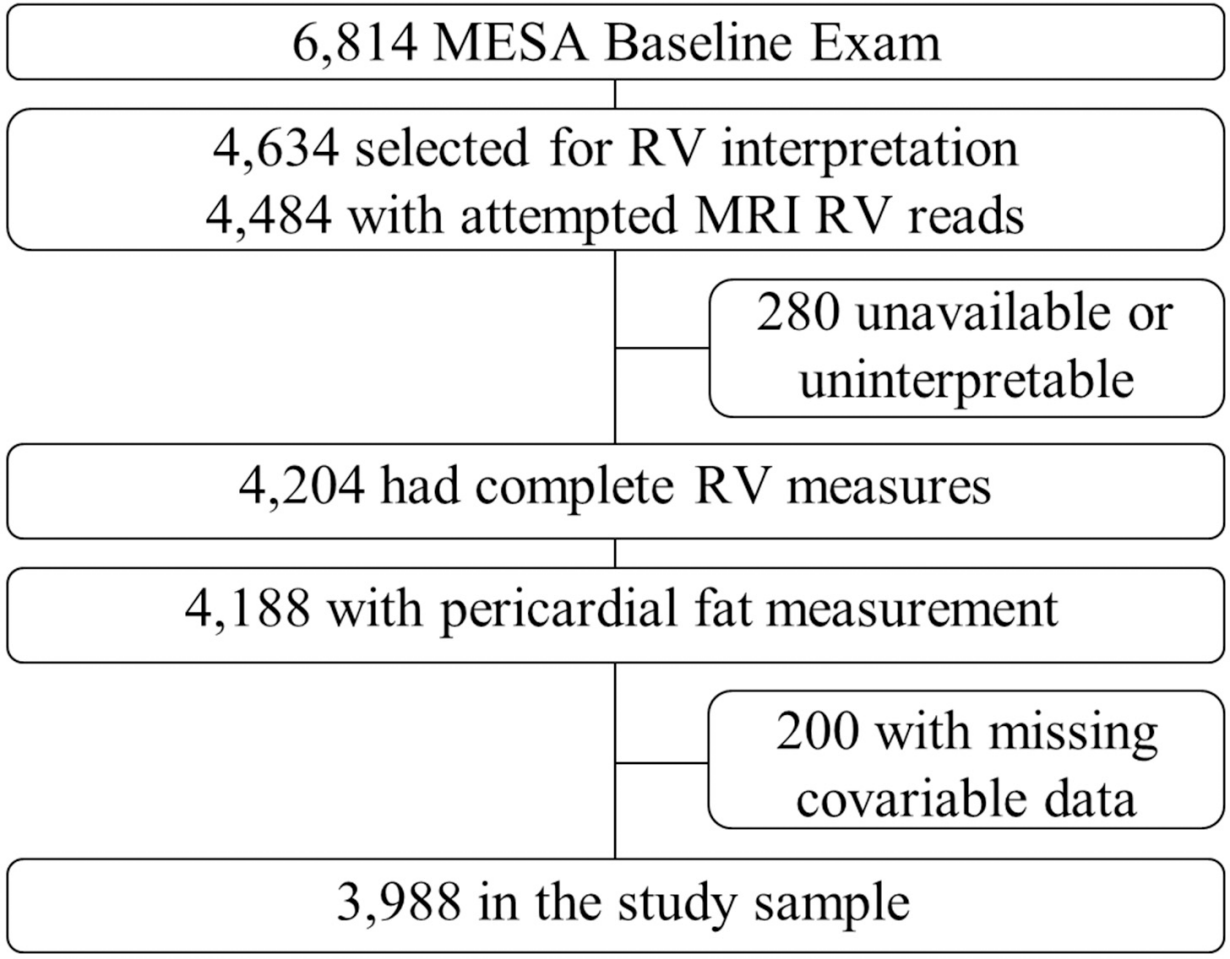
Study sample. MESA = Multi- Ethnic Study of Atherosclerosis; MRI = magnetic resonance imaging; RV = right ventricle.

MESA enrolled 6,814 participants. Cardiac MRI reads were attempted in 4,484 participants before achieving the study goal of 4,204 participants (94% of attempted reads) (Fig 1). Pericardial fat was measured in 4,188 (99%) of these participants by CT. Two hundred participants (4.7%) were excluded because of missing self-reported income (n = 128), education (n = 14), pack years of smoking (n = 47), weekly exercise (n = 2), or incomplete measures used to define the metabolic syndrome (n = 9). The final study sample included 3,988 participants (Fig 1). The mean age was 61.3 years and 53.0% were women (Table 1). Mean RV mass in the study sample was 21.1g SD 4.4g, mean RV-EDV was 124.1mL SD 30.8mL, mean RV-ESV was 37.2mL SD 14.2, mean RV-SV 86.9mL SD 20.5mL, and mean RV-EF was 70.5% SD 6.4%. Mean pericardial fat was 76.7cm^3^ SD 39.5 cm^3^.

**Table 1:** Characteristics of the study sample.

	Study Sample (n = 3988)		Excluded (n = 2826)
Age, years	61.3 (10.1)	63.3 (10.4)	
Female, %	53	53	
Race, %			
White	40	37	
African American	13	11	
Hispanic	25	31	
Chinese	22	22	
Education, %			
No high school degree	9	13	
High school degree	25	26	
Some college	16	17	
Bachelors or advanced degree	50	44	
Income previous 12 months, %			
<$25,000	30	34	
$25–50,000	30	27	
$50–100,000	27	26	
>$100,000	14	13	
Height, cm	166.4 (9.9)	166.3 (10.2)	
Waist Circumference, cm	96.7 (13.4)	100.1 (15.5)	
Hypertension, %	42.4	48.4	
Systolic blood pressure, mmHg	125.3 (20.9)	128.4 (22.1)	
Diastolic blood pressure, mmHg	71.8 (10.1)	71.9 (10.3)	
Impaired Fasting glucose, %	13.1	14.7	
Untreated Diabetes	2.3	3.0	
Treated Diabetes	9.1	11.1	
Total cholesterol, mg/dl	194.3 (35)	193.8 (36.7)	
High-density lipoprotein, mg/dl	51.1(15)	50.7 (14.6)	
Total Trigylceride, mg/dl	131.1 (85.4)	132.3 (93.5)	
Presence of Metabolic Syndrome, %	34	39	
Smoking status, %			
Never smoker	52.6	47.0	
Former smoker	35.0	38.9	
Current Smoker	12.4	14.1	
Pack years, among ever smokers	10.7 (20.6)	12.3 (21.3)	
Exercise reported, mets/wk ^*^	1611 (2409)	1470 (2236)	
CRP, mg/L	3.5 (5.5)	4.2 (6.4)	
IL-6, pg/ml	1.5 (1.2)	1.7 (1.3)	
Pericardial fat, cm^3^	76.7 (39.5)	79.5 (41.2)	

*1 metabolic equivalent = the rate of energy produced per unit surface area of a person seated at rest. Results reported as a percent or mean (standard deviation) as appropriate

**Fig 2:**
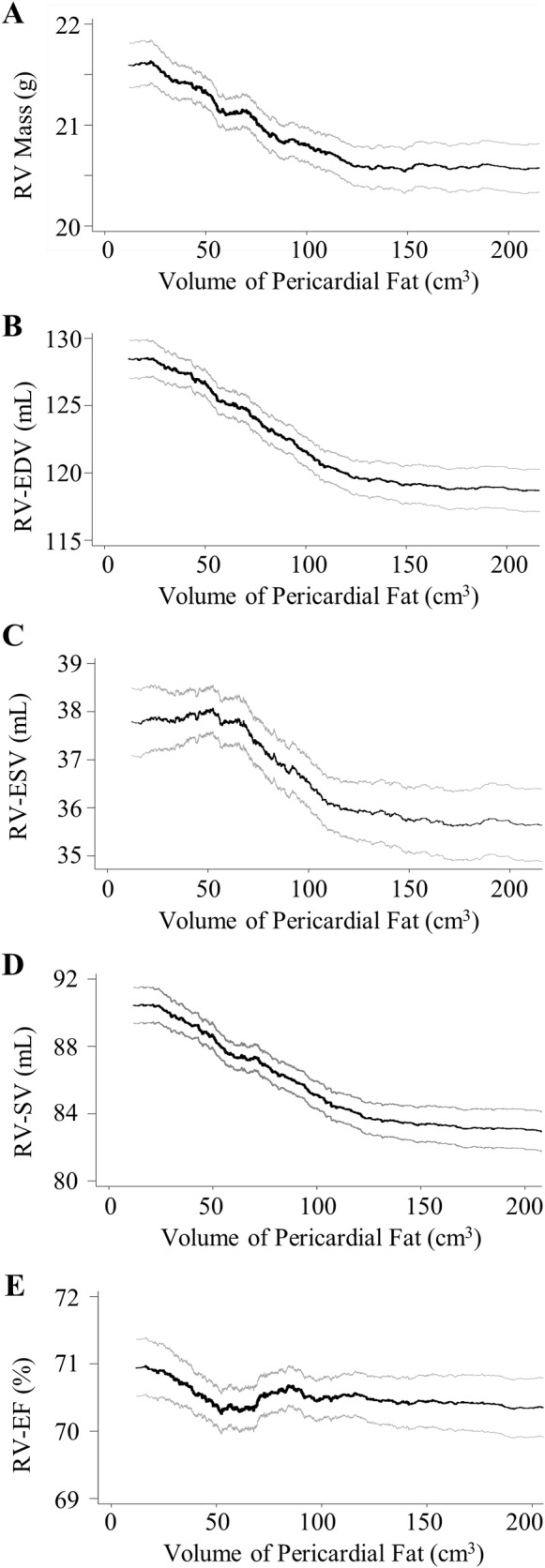
Multivariable nonparametric smoothed relationship between pericardial fat volume in cm3 and right ventricular (RV) parameters with adjustment for age, gender, race, height, weight, study site, education level, exercise habits, and smoking status including pack years smoked (black line). Gray lines represent 95% Confidence intervals. The relationship between pericardial fat and (A) RV mass, (B) RV end diastolic volume (EDV), (C) RV end systolic volume, (D) RV stroke volume (SV), (E) ejection fraction (EF).

Greater pericardial fat volume was associated with greater RV mass (0.5 g for a 40 cm^3^ increase in pericardial fat volume, p <0.001) without adjustment for weight. After adjusting for weight (which was expected to be a confounder), the relationship reversed, so that at any given weight, greater pericardial fat volume was associated with significantly *lower* RV mass (-0.3 g for a 40 cm^3^ increase in pericardial fat volume, p <0.001). This inverse relationship was unchanged after accounting for differences in socioeconomic status and cardiovascular health behaviors (Table 2 and Fig 2). The relationship remained after adjustment for the presence or absence of the metabolic syndrome, for markers of systemic inflammation, and for left ventricular mass (all p <0.001) (Table 3).

**Table 2:** Multivariable linear regression estimating associations between pericardial fat and right ventricular structure and function (n = 3,988).

	Per 40 cm3 increase in Pericardial fat		
	*Difference (*β *coefficient)*	95% CI	*p-value*
RV mass, g			
Limited adjustment^*^	0.5	0.4 to 0.6	<0.001
Limited adjustment with weight	-0.3	-0.5 to -0.2	<0.001
Full Adjustment^†^	-0.3	-0.4 to -0.2	<0.001
RV-EDV, mL			
Limited adjustment	2.4	1.7 to 3.2	<0.001
Limited adjustment with weight	-3.7	-4.6 to -2.8	<0.001
Full Adjustment	-3.4	-4.3 to -2.5	<0.001
RV-ESV, mL			
Limited adjustment	1.0	0.6 to 1.4	<0.001
Limited adjustment with weight	-1.0	-1.5 to -0.6	<0.001
Full Adjustment	-1.0	-1.4 to -0.5	<0.001
RV-SV, mL			
Limited adjustment	1.4	0.9 to 2.0	<0.001
Limited adjustment with weight	-2.7	-3.3 to -2.0	<0.001
Full Adjustment	-2.4	-3.1 to -1.8	<0.001
RV-EF, %			
Limited adjustment	-0.3	-0.5 to -0.1	0.01
Limited adjustment with weight	-0.1	-0.3 to 0.2	0.55
Full Adjustment	-0.1	-0.3 to 0.2	0.59

Abbreviations: RV = right ventricle; EDV = end-diastolic volume; ESV = end-systolic volume; SV = stroke volume; EF = ejection fraction; CI = confidence interval

* Limited model: age, sex, race/ethnicity, height, and study site

† Full model: Limited with weight + education level, income, exercise habits, smoking status, and pack-years

**Table 3:** Multivariable linear regression estimating associations between pericardial fat and right ventricular structure and function after accounting for metabolic syndrome, markers of systemic inflammation (CRP & IL-6), and in left ventricular morphology (n = 3,988).

	Per 40 cm3 increase in Pericardial fat			
	*Difference (*β *coefficient)*	95% CI		*p-value*
RV mass, g				
Full Adjustment^*^ + Metabolic syndrome^†^	-0.3	-0.4 to -0.2	<0.001	
Full Adjustment + CRP and IL-6	-0.3	-0.4 to -0.2	<0.001	
Full adjustment + LV Mass	-0.3	-0.4 to -0.1	<0.001	
RV-EDV, mL				
Full Adjustment + Metabolic syndrome	-3.2	-4.1 to -2.3	<0.001	
Full Adjustment + CRP and IL-6	-3.3	-4.2 to -2.4	<0.001	
Full Adjustment + LV-EDV	-0.8	-1.5 to -0.2	0.01	
RV-ESV, mL				
Full Adjustment + Metabolic syndrome	-0.8	-1.3 to -0.4	<0.001	
Full Adjustment + CRP and IL-6	-0.9	-1.3 to -0.4	<0.001	
Full Adjustment + LV-ESV	-0.4	-0.8 to 0	0.04	
RV-SV, mL				
Full Adjustment + Metabolic syndrome	-2.4	-3.1 to -1.7	<0.001	
Full Adjustment + CRP and IL-6	-2.4	-3.1 to -1.7	<0.001	
Full Adjustment + LV mass	-2.2	-2.8 to -1.6	<0.001	
RV-EF, %				
Full Adjustment + Metabolic syndrome	-0.1	-0.4 to 0.1	0.35	
Full Adjustment + CRP and IL-6	-0.1	-0.3 to 0.2	0.50	
Full Adjustment + LV-EF	-0.1	−0.3 to 0.1	0.34	

Abbreviations: RV = right ventricle; LV = left ventricle; EDV = end-diastolic volume; ESV = end-systolic volume; SV = stroke volume; EF = ejection fraction; CRP = C-reactive protein; IL-6 = interleukin-6; CI = confidence interval

* Full model: Limited with weight + education level, income, exercise habits, smoking status, and pack years

† Metabolic syndrome (2001 NCEP Adult Treatment Panel III guidelines) was present when 3 or more of the following criteria met: (1) Waist circumference >102cm for men or >88cm for women, (2) triglycerides >150 mg/dL, (3) HDL < 40mg/dl for men or <50 mg/dl for women, (4) blood pressure ≥ 135/85mmHg, (5) fasting plasma glucose ≥ 110 mg/dl

Greater pericardial fat volume was associated with a larger RV-EDV (2.4 mL for a 40 cm^3^ increase in pericardial fat volume, p<0.001) without adjustment for weight. After adjusting for weight the relationship again reversed, and so that among individuals of similar weight, greater pericardial fat volume was associated with a smaller RV-EDV (-3.7ml for a 40 cm^3^ increase in pericardial fat volume, p<0.001). This inverse relationship was unchanged after accounting for differences in socioeconomic status, cardiovascular health behaviors, markers of systemic inflammation, and the presence or absence of the metabolic syndrome (n = 3,988, all p< 0.01) (Tables 2 and 3, Fig 2). The relationship between pericardial fat and RV-EDV was attenuated (but still statistically significant) when adjusted for LV-EDV (-0.8 mL, p = 0.01) (Table 3).

Greater pericardial fat volume was associated with a larger RV-ESV (1.0 mL for a 40 cm^3^ increase in pericardial fat volume, p<0.001) without adjustment for weight. After adjusting for weight, the relationship reversed. Among individuals of similar weight, greater pericardial fat volume was associated with a smaller RV-ESV (-1.0ml for a 40 cm^3^ increase in pericardial fat volume, p<0.001). This inverse relationship was unchanged after accounting for differences in socioeconomic status, cardiovascular health behaviors, markers of systemic inflammation, and the presence or absence of the metabolic syndrome (n = 3,988, all p< 0.01) (Tables 2 and 3, Fig 2). The relationship between pericardial fat and RV-ESV was attenuated (but still statistically significant) when adjusted for LV-ESV (-0.4 mL, p = 0.04) (Table 3).

Greater pericardial fat volume was associated with larger RV-SV (1.4 mL for a 40 cm^3^ increase in pericardial fat volume) without adjustment for weight. After adjusting for weight, the relationship reversed, and among individuals of otherwise similar weight, greater pericardial fat volume was associated with significantly smaller RV-SV (-2.7 mL for a 40 cm^3^ increase in pericardial fat volume, p = <0.001). This inverse relationship was unchanged after accounting for differences in socioeconomic status and cardiovascular health behaviors (Table 2 and Fig 2). The relationship remained after adjustment for the presence or absence of the metabolic syndrome, for markers of systemic inflammation, and for left ventricular mass (all p <0.001) (Table 3).

No association was seen between pericardial fat and RV-EF. The lack of a relationship was not changed with further adjustment for differences in socioeconomic status, cardiovascular health behaviors, LV function, the presence or absence of the metabolic syndrome, or markers of systemic inflammation (Tables 2 and 3, Fig 2).

In sensitivity analyses adjusting for components of the metabolic syndrome rather than the overall presence or absence of the metabolic syndrome (full adjustment with further adjustment for waist circumference, triglycerides, HDL, systolic and diastolic blood pressure, and fasting plasma glucose) results were unchanged. There was no difference in the relationships between pericardial fat volume and RV mass, EDV, ESV, SV or EF relative to that seen with simply adjusting for a composite variable accounting for the metabolic syndrome (S1 Table). In exploratory models, age, gender, the presence of diabetes or the metabolic syndrome were not found to be effect modifiers of the association between pericardial fat and RV morphology (all p for the interaction > 0.05).

## Discussion

We have shown that greater pericardial fat volume is associated with lower RV mass, smaller RV-EDV, smaller RV-ESV, and a smaller RV-SV in a multi-ethnic, multi-city cohort of adults without clinical cardiovascular disease after adjustment for age, gender, weight, and other covariates. The magnitude of observed difference in RV mass over the range of pericardial fat are similar to the magnitude of difference in LV mass between men and women with and without diabetes or those who do and do not smoke [18,19], which may support the biologic relevance of our observations.

Pericardial fat is strongly linked to weight in MESA participants [14] and RV mass, RV-EDV, RV-ESV, and RV-SV increase with increases in weight and body mass index [20]. These changes in RV morphology may be attributable to increases in blood volume, increases in RV afterload from sleep disordered breathing, changes in adipokine levels, and fatty infiltration of the RV. Therefore, it is not surprising that pericardial fat is associated with a greater RV mass, a larger RV-EDV, a larger RV-ESV, and a larger RV-SV in models without adjustment for weight. However, our findings suggest that among individuals of otherwise similar weight pericardial fat has a relationship with cardiovascular morphology that is distinct and opposite from that seen with increased weight alone.

Previous work suggests our findings could be explained by differences in myocardial inflammation attributed to pericardial fat. Pericardial fat is thought to be a potent paracrine reservoir of inflammatory mediators immediately adjacent to the RV myocardium [8]. We have shown that elevated markers of systemic inflammation (as estimated by CRP, IL-6, MMP9, and TNF-R1) are associated with reduced RV mass, a smaller RV-EDV, a smaller RV-ESV, and a smaller RV-SV in MESA participants [7,21]. Inflammation likely contributes to differences in RV myocardial biology through multiple mechanisms including dysregulation of cardiac myocyte apoptosis, through increases in unopposed reactive oxygen species, and through reduction in cardiac myocyte contractility, which may combine to promote RV diastolic dysfunction [22]. We speculate that the thin walled right ventricle is susceptible to decreases in myocardial compliance imposed by inflammation and fibrosis. Given the low pressure generated by the normal RV, a decrease in compliance would likely lead to a decrease in size. It is notable that relationships between pericardial fat volume and RV morphology did not change after accounting for systemic levels of CRP and IL-6 in this study. This suggests that, if inflammation is important to the relationship between pericardial fat and RV morphology as we suspected a priori, it is through an inflammatory pathway that does not involve CRP and IL-6 or it is a paracrine and not a systemic mediator of inflammation that drives the association.

Pericardial fat may also be a marker of the metabolic syndrome [23]. Metabolic syndrome is related to myocardial adaptation, coronary vascular disease, stroke and cardiovascular related mortality [24]. While it would have been plausible that pericardial fat was related to RV morphology merely as a marker of the metabolic syndrome, our data does not support this. The presence or absence of metabolic syndrome neither confounded nor was an effect modifier for the relationship between pericardial fat and any of the RV parameters evaluated.

Previous studies have demonstrated that pericardial fat volume is associated with left ventricular morphology [3,4, 25]. In the largest single analysis of pericardial fat and left ventricular morphology, pericardial fat was positively correlated with LV mass and LV-EDV on cardiac MRI when the authors did not account for weight or systemic adiposity [25]. Our findings in the RV are similar, as RV mass and RV-EDV increase alongside increases in pericardial fat when a participant’s weight is not considered. Our results in the RV diverge from the description of pericardial fat and the LV when weight *is* considered. In the LV, there does not appear to be a relationship between pericardial fat and LV morphology when weight or systemic adiposity is considered [25]. However, in the RV, our results suggest the relationship reverses, but remains statistically significant when weight is considered. Although in some cases it is attenuated, the inverse relationship between pericardial fat and the RV persists with adjustment for LV morphology, suggesting relationships between pericardial fat and the RV are largely independent of the LV. Differences between the LV and the RV are well established and likely reflect the distinct fiber orientation, embryology, hemodynamic loading conditions, and morphology of the RV relative to the LV [26]. This has several practical implications, and there is precedent that inflammation, afterload, and cardiac stress impact the right and left ventricles differently [27].

Because participants with clinical cardiovascular disease were excluded from MESA, none of our study subjects had pulmonary arterial hypertension; however, pulmonary arterial hypertension is a prototypic disease of right heart failure and our findings may have clinical application to this disease. It is speculative; however plausible, that factors related to right heart morphology in individuals without cardiovascular disease may be relevant to right heart adaptation in the setting of cardiopulmonary disease. Increased pericardial fat volume has been associated with elevated systolic pulmonary arterial pressure [28]. Our data join this observation to suggest that pericardial fat, a common feature in overweight individuals [4], may be relevant to right ventricular myocardial adaptation. Pericardial fat, therefore, may be related both to the presence of pulmonary arterial hypertension and to RV morphology. This paradigm could be further explored in participants with pulmonary arterial hypertension.

This study has limitations. Our study was observational. As a result, residual or unmeasured confounding is possible, temporality is unknown, and causality cannot be confirmed. It is reassuring that adjustment for several potential confounders did not change the relationship; however, cautious inference is required. We have found an inverse relationship between pericardial fat and RV morphology; however, our discussion of mechanism is speculative. Also, our pericardial fat measurements included two different types of fat with unique origins; however, correlation between pericardial fat and epicardial fat was high (0.92) and we anticipate misclassification using this approach to be small [15]. Similarly, prior studies have suggested RV pericardial fat and LV pericardial fat, when analyzed separately, may more accurately reflect the relationship between cardiac adipose tissue and the underlying myocardium. We did not measure this distinction in the current study, but this could be considered in the future investigation.

Finally, relevance to clinical disease is thought provoking, but speculative. Unfortunately, we do not know what change in RV parameters are “clinically significant” especially in individuals without clinical cardiovascular disease. We mention pulmonary hypertension, but our intent is not to develop disease predictors; instead, our intent is to identify associations that may hint at relevant mechanisms in RV myocardial biology and adaptation. While we cannot be certain what magnitude of association meets this criterion, in the non-diseased heart these associations may be small. A two standard deviation change in pericardial fat is associated with ~5% change in RV-EDV. This magnitude exceeds the difference in LV-EDV in MESA participants with and without diabetes and is similar to that seen in participants whose blood pressure differs by 21 mmHg [29,30]. Both hypertension and diabetes are known to be mechanistically important to the health and adaptation of the left heart and this context may support the relevance of our findings [30].

## Conclusions

After adjusting for weight, greater pericardial fat volume was associated with reduced RV mass, a smaller RV-EDV, a smaller RV-ESV, and a smaller RV-SV. This relationship was independent of socioeconomic status, cardiovascular risk factors, the presence or absence of the metabolic syndrome, and differences in markers of systemic inflammation. The relationship between pericardial fat and RV morphology could be relevant to diseases of right heart failure.

## Supporting Information

S1 Table Multivariable linear regression estimating associations between pericardial fat and right ventricular structure and function with adjustment for composite variable of Metabolic Syndrome and for individual components of Metabolic Syndrome to assess for residual confounding (n = 3988). (DOC)
