# Supplementary material for: Pericardial Fat and Right Ventricular Morphology: The Multi-Ethnic Study of Atherosclerosis- Right Ventricle Study (MESA-RV)
Source: PLoS One. 2016 Jun 16;11(6):e0157654. doi: 10.1371/journal.pone.0157654 (PMC4911142; doi:10.1371/journal.pone.0157654)
Supplement: S1 Table — (DOC) [file pone.0157654.s001.doc]

**Pericardial Fat and Right Ventricular Morphology: The Multi-Ethnic Study of Atherosclerosis- Right Ventricle Study (MESA-RV)**

David S. Wenger, MD; Steven M. Kawut, MD, MS; Jingzhong Ding, MD; David A. Bluemke, MD, PhD; Catherine L Hough, MD, MS; Richard A. Kronmal, PhD; Joao A. Lima, MD; Peter J. Leary, MD, MS

**Online Supplement**

**S1 Table. Multivariable linear regression estimating associations between pericardial fat and right ventricular structure and function with adjustment for composite variable of Metabolic Syndrome and for individual components of Metabolic Syndrome to assess for residual confounding (n=3988)**

|  | **Per 40 cm3 increase in Pericardial fat** | | | | |
| --- | --- | --- | --- | --- | --- |
|  |  |  | *Difference (*β *coefficient)* | 95% CI | *p* value |
| | RV mass, g | | --- | |  |  |  |  |  |
| Full Adjustment* + MS Composite‡ |  |  | -0.3 | -0.4 to -0.2 | <0.001 |
| Full Adjustment +individual components of MSˠ | |  | -0.2 | -0.3 to -0.1 | 0.005 |
|  |  |  |  |  |  |
| RV-EDV, mL |  |  |  |  |  |
| Full Adjustment + MS Composite |  |  | -3.2 | -4.1 to -2.3 | <0.001 |
| Full Adjustment +individual components of MS | |  | -2.6 | -3.3 to -1.6 | <0.001 |
|  |  |  |  |  |  |
| RV-ESV, mL |  |  |  |  |  |
| Full Adjustment + MS Composite |  |  | -0.8 | -1.3 to -0.4 | <0.001 |
| Full Adjustment +individual components of MS | |  | -0.9 | -1.4 to -0.4 | <0.001 |
|  |  |  |  |  |  |
| RV-SV, mL |  |  |  |  |  |
| Full Adjustment + MS Composite |  |  | -2.4 | -3.1 to -1.7 | <0.001 |
| Full Adjustment +individual components of MS |  |  | -1.8 | -2.4 to -1.1 | <0.001 |
|  |  |  |  |  |  |
| RV-EF, % |  |  |  |  |  |
| Full Adjustment + MS Composite |  |  | -0.1 | -0.4 to 0.1 | 0.3 |
| Full Adjustment +individual components of MS |  |  | -0.1 | -0.3 to 0.2 | 0.7 |

Abbreviations: MS= Metabolic Syndrome; EDV= end-diastolic volume; ESV= end-systolic volume; SV= stroke volume; EF= ejection fraction

*Full model: Limited +education level, income, exercise habits, smoking status, and pack years

‡ Metabolic syndrome (2001 NCEP Adult Treatment Panel III guidelines) was present when 3 or more of the following criteria met: (1) Waist circumference >102cm for men or >88cm for women, (2) triglycerides >150 mg/dL, (3) HDL < 40mg/dl for men or <50 mg/dl for women, (4) blood pressure ≥ 135/85mmHg, (5) fasting plasma glucose ≥ 110 mg/dl

ˠ Adjustment for waist circumference, triglycerides, HDL cholesterol, systolic blood pressure, diastolic blood pressure, and fasting plasma glucose
